# Sonographic Images of Hepatic Portal Venous Gas in a Patient with Gastrointestinal Ischemia

**DOI:** 10.3390/diagnostics12092034

**Published:** 2022-08-23

**Authors:** Piotr F. Czempik, Oskar Bożek, Łukasz J. Krzych

**Affiliations:** 1 Department of Anaesthesiology and Intensive Care, Faculty of Medical Sciences in Katowice, Medical University of Silesia, 40-752 Katowice, Poland; 2 Department of Radiodiagnostics and Invasive Radiology, Faculty of Medical Sciences in Katowice, Medical University of Silesia, 40-752 Katowice, Poland

**Keywords:** liver ultrasound, hepatic portal venous gas, mesenteric ischemia

## Abstract

Hepatic portal venous gas (HPVG) detected by ultrasound (US) following liver transplantation or in suppurative cholangitis was described previously. To our knowledge, there have been no descriptions of HPVG detected by US in acute mesenteric ischemia. Here we present diagnostic images of a 52-year-old female who was admitted to the intensive care unit (ICU) following successful embolization of a ruptured saccular aneurysm of the right vertebral artery. During their stay in the ICU, the patient developed hypotension with low systemic vascular resistance and hypovolemia. Based on physical examination of the abdomen and laboratory results, preliminary diagnosis of intra-abdominal sepsis was made. Early abdominal US was performed to find the source of sepsis. The preliminary diagnosis of stomach/small intestine ischemia was made by ultrasonic detection of HPVG. Other less likely diagnoses were pneumobilia due to cholangitis, hepatic micro-abscesses, and punctuate calcifications. The diagnosis was confirmed by multi-phase abdominal computed tomography. The explorative laparotomy revealed necrosis of the stomach, small intestine, and liver. Due to the severity of necrosis, surgical treatment was abandoned. Provided sonographic images show HPVG as an ominous sign of small intestine and stomach ischemia. Early liver US should be performed whenever intra-abdominal pathology is suspected.

**Figure 1:**
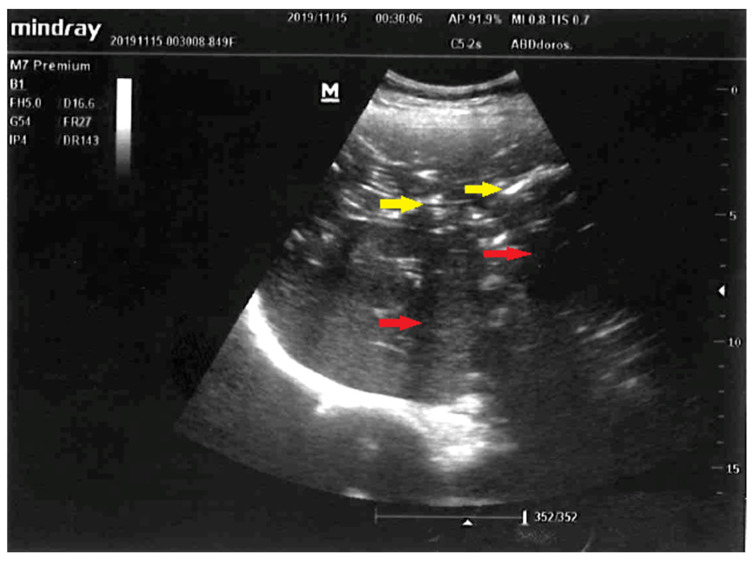
Early liver ultrasound (US) performed to find a source of sepsis in a 52-year-old female suspected to have developed intraabdominal sepsis. Visualized here are: normal-sized liver, punctuated hyperechoic foci with anti-gravitational distribution (yellow arrows) casting acoustic shadows (red arrows). Due to characteristic appearance on liver US, the most likely diagnosis was hepatic portal venous gas (HPVG) [1,2]. Other less likely diagnoses were: pneumobilia due to cholangitis, hepatic micro-abscesses, and punctuate calcifications. Hepatic portal venous gas detected by US following liver transplantation or in suppurative cholangitis were described previously [3,4]. Although presence of mesenteric ischemia and gas in the portal venous circulation was previously revealed with computed tomography [5,6], in our case early abdominal US was performed, what expedited definite diagnosis and targeted treatment: choice of empiric broad-spectrum antibiotics and explorative laparotomy. This is the first time, to the best of our knowledge, that HPVG was visualized with US in the setting of acute mesenteric ischemia.

**Figure 2:**
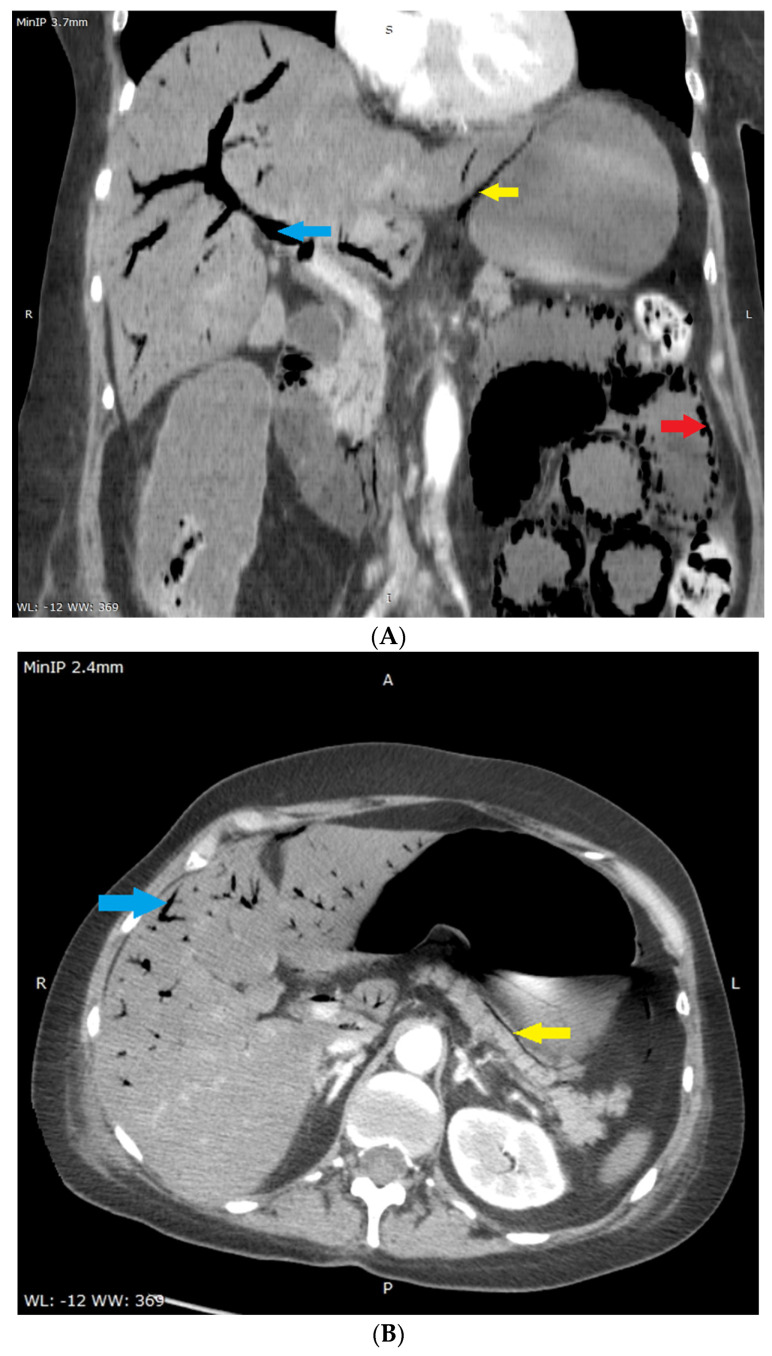
(**A**,**B**) Multi-phase abdominal computed tomography performed immediately after liver US. No oral contrast was administered due to urgency–the stomach is filled with accidental content making it isodense to the liver parenchyma. Therefore it is difficult to distinguish this from liver parenchyma in (**A**). Routine amount of intravenous contrast agent was administered. (**A**)–coronal reconstruction, portal-venous phase, slice showing the portal vein was selected, minimum intensity projection with 3.7 mm thickness was used in order to better show gas in the portal circulation. Visualized are: gas in the wall of the small intestine (red arrow), gas in the portal vein (blue arrow) and most of its branches-on the picture visible mostly in segments V and VIII, gas in the wall of stomach-lesser curvature (yellow arrow), and distended small intestine filled with fluid (width 33 mm). (**B**)–axial plane, portal-venous phase, at the level of pancreas, adrenal glands and portal vein, minimum intensity projection with 2.4 mm thickness was used for the same reason as in the (**A**). Visualized are: gas in the portal vein (visible gas-blood level) and most of its branches (blue arrow), paring of hepatic segment VI portal branches due to anti-gravitational distribution, gas in the wall of stomach (yellow arrow). No gas was present in the hepatic vein circulation as an aquarium sign [7], the celiac trunk and superior mesenteric artery were narrow but patent, there were single gas microbubbles present in the spleen and in superior mesenteric vein, intra- and extrahepatic ducts were not dilated, and no pneumobilia was present.

**Figure 3:**
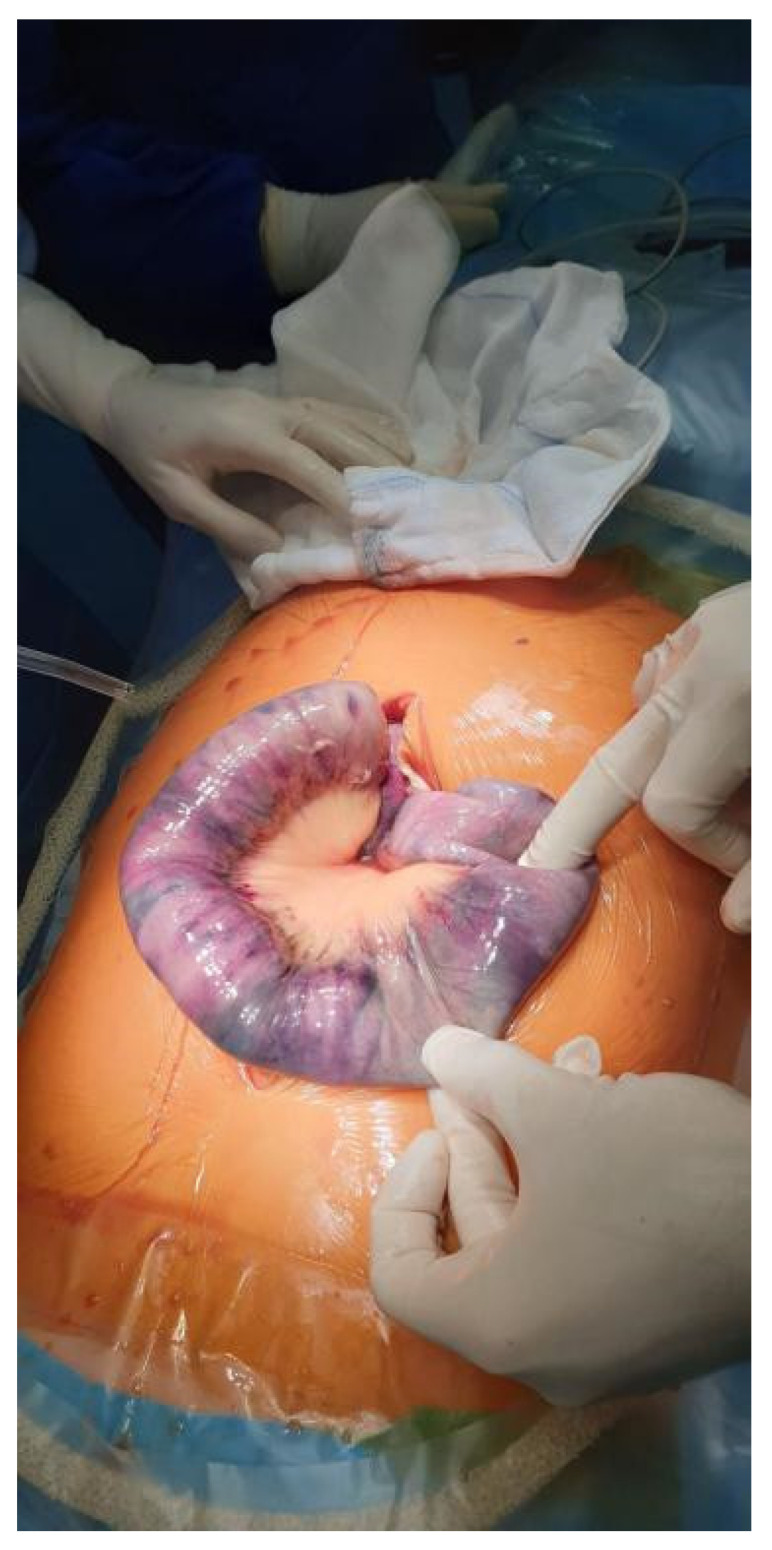
Intraoperative image taken during an explorative laparotomy. The photograph reveals necrosis of the stomach and small intestine. Due to the severity of necrosis, surgical treatment was abandoned. The patient died the following day from multi-organ failure.

## Data Availability

Not applicable.
